# (*E*,*E*)-*N*,*N*′-Bis[4-(methyl­sulfon­yl)benzyl­idene]ethane-1,2-diamine

**DOI:** 10.1107/S1600536809047527

**Published:** 2009-11-14

**Authors:** Shao-Song Qian, Hong-You Cui

**Affiliations:** a School of Life Sciences, ShanDong University of Technology, ZiBo 255049, People’s Republic of China; b School of Chemical Engineering, ShanDong University of Technology, ZiBo 255049, People’s Republic of China

## Abstract

In the crystal structure of the title Schiff base compound, C_18_H_20_N_2_O_4_S_2_, the mol­ecule lies across a crystallographic inversion centre. The torsion angle of the N—C—C—N fragment is 180°, as the inversion centre bis­ects the central C—C bond. The crystal packing is stabilized by C—H⋯O hydrogen bonds and aromatic π–π stacking inter­actions with a centroid–centroid distance of 3.913 (2) Å.

## Related literature

For bond-length data, see: Allen *et al.* (1987 ▶); For the crystal structure of a similar Schiff base compound, see: Sun *et al.* (2004 ▶). For the crystal structure of a precursor mol­ecule used in the synthesis of the title compound, see: Qian & Cui (2009 ▶).
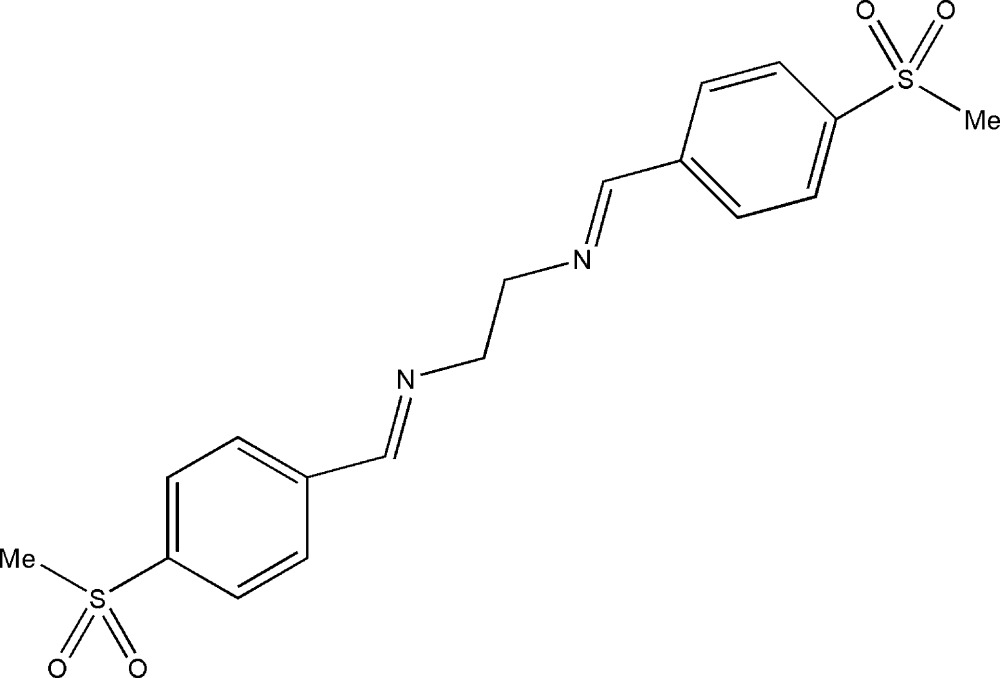


## Experimental

### 

#### Crystal data

C_18_H_20_N_2_O_4_S_2_
*M* *_r_* = 392.48
Triclinic, 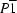

*a* = 7.0100 (14) Å
*b* = 8.0530 (16) Å
*c* = 8.8740 (18) Å
α = 88.06 (3)°
β = 67.56 (3)°
γ = 87.60 (3)°
*V* = 462.53 (19) Å^3^
*Z* = 1
Mo *K*α radiation
μ = 0.31 mm^−1^
*T* = 293 K
0.20 × 0.10 × 0.10 mm

#### Data collection

Enraf–Nonius CAD-4 diffractometer
Absorption correction: multi-scan (*SHELXTL*; Sheldrick, 2008 ▶) *T*_min_ = 0.940, *T*_max_ = 0.969
1830 measured reflections
1683 independent reflections
1374 reflections with *I* > 2σ(*I*)
*R*_int_ = 0.017
3 standard reflections every 200 reflections intensity decay: 1%

#### Refinement

*R*[*F* ^2^ > 2σ(*F* ^2^)] = 0.045
*wR*(*F* ^2^) = 0.153
*S* = 1.00
1683 reflections
118 parameters
H-atom parameters constrained
Δρ_max_ = 0.20 e Å^−3^
Δρ_min_ = −0.33 e Å^−3^

### 

Data collection: *CAD-4 Software* (Enraf–Nonius, 1989 ▶); cell refinement: *CAD-4 Software*; data reduction: *XCAD4* (Harms & Wocadlo, 1995 ▶); program(s) used to solve structure: *SHELXS97* (Sheldrick, 2008 ▶); program(s) used to refine structure: *SHELXL97* (Sheldrick, 2008 ▶); molecular graphics: *SHELXTL* (Sheldrick, 2008 ▶); software used to prepare material for publication: *SHELXTL*.

## Supplementary Material

Crystal structure: contains datablocks global, I. DOI: 10.1107/S1600536809047527/wm2281sup1.cif

Structure factors: contains datablocks I. DOI: 10.1107/S1600536809047527/wm2281Isup2.hkl

Additional supplementary materials:  crystallographic information; 3D view; checkCIF report

## Figures and Tables

**Table 1:** Hydrogen-bond geometry (Å, °)

*D*—H⋯*A*	*D*—H	H⋯*A*	*D*⋯*A*	*D*—H⋯*A*
C4—H4*A*⋯O1^i^	0.93	2.52	3.241 (4)	135

Symmetry code: (i) 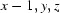.

**Fig. 1.**
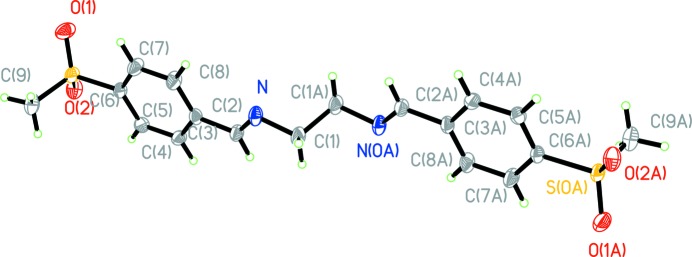
The structure of (I), showing the atom-labelling scheme. Displacement ellipsoids are drawn at the 35% probability level. The molecule is completed by symmetry operation (-x, -y, 2-z) across the central C—C bond.

**Fig. 2.**
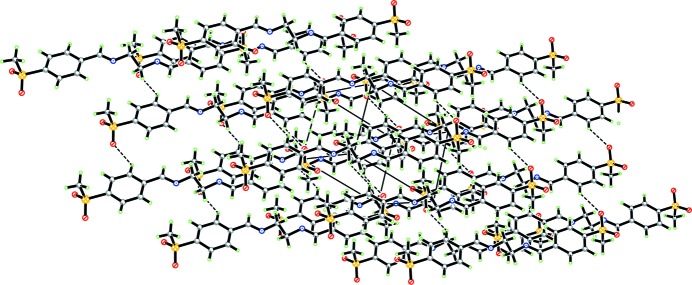
Plot of the crystal packing of compound (I). C—H···O hydrogen bonds are indicated with dotted lines.

## supplementary crystallographic information

### Comment

The title compound, (I), acts as an important precursor for the synthesis of Schiff base complexes. As an extension of our work on the structural characterization of Schiff base compounds, the crystal structure is reported here.

The asymmetric unit contains one-half of the molecule of (I), the other half being inversion-related by symmetry operation (-x, -y, 2-z) (Fig.1). All the bond lengths are within normal ranges (Allen *et al.*, 1987) and comparable to the values observed in other similar compounds (Qian & Cui, 2009; Sun *et al.*, 2004). The crystal packing is stabilized by C—H···O hydrogen bonds and aromatic π-π stacking interactions with a centroid-centroid distance of 3.913 (2) Å (Figure 2, Table 1). The torsion angle of the N—C—C—N fragment is 180 °, as the inversion centre bisects the central C—C bond.

### Experimental

4-(methylsulfonyl)benzaldehyde (0.184 g, 1 mmol) (Qian & Cui, 2009) and ethylene diamine (0.03 g, 0.5 mmol) were dissolved in acetonitrile (20 ml). The mixture was stirred at room temperature for 10 min to give a clear yellow solution. After keeping the solution in air for 10 d, yellow block-shaped crystals of (I) were formed at the bottom of the vessel on slow evaporation of the solvent.

### Refinement

All H atoms were placed in geometrical positions and constrained to ride on their parent atoms with C—H distances in the range 0.93–0.96 Å, They were treated as riding atoms, with *U*_iso_(H) = k*U*_eq_(C), where k = 1.5 for methyl and 1.2 for all other H atoms.

### Crystal data

**Table:**

C_18_H_20_N_2_O_4_S_2_	*Z* = 1
*M**_r_* = 392.48	*F*(000) = 206
Triclinic, *P*1	*D*_x_ = 1.409 Mg m^−^^3^
Hall symbol: -P 1	Mo *K*α radiation, λ = 0.71073 Å
*a* = 7.0100 (14) Å	Cell parameters from 25 reflections
*b* = 8.0530 (16) Å	θ = 9–13°
*c* = 8.8740 (18) Å	µ = 0.31 mm^−^^1^
α = 88.06 (3)°	*T* = 293 K
β = 67.56 (3)°	Block, yellow
γ = 87.60 (3)°	0.20 × 0.10 × 0.10 mm
*V* = 462.53 (19) Å^3^	

### Data collection

**Table:**

Enraf–Nonius CAD-4 diffractometer	1374 reflections with *I* > 2σ(*I*)
Radiation source: fine-focus sealed tube	*R*_int_ = 0.017
graphite	θ_max_ = 25.3°, θ_min_ = 2.5°
ω/2θ scans	*h* = 0→8
Absorption correction: multi-scan (*SHELXTL*; Sheldrick, 2008)	*k* = −9→9
*T*_min_ = 0.940, *T*_max_ = 0.969	*l* = −9→10
1830 measured reflections	3 standard reflections every 200 reflections
1683 independent reflections	intensity decay: 1%

### Refinement

**Table:**

Refinement on *F*^2^	Primary atom site location: structure-invariant direct methods
Least-squares matrix: full	Secondary atom site location: difference Fourier map
*R*[*F*^2^ > 2σ(*F*^2^)] = 0.045	Hydrogen site location: inferred from neighbouring sites
*wR*(*F*^2^) = 0.153	H-atom parameters constrained
*S* = 1.00	*w* = 1/[σ^2^(*F*_o_^2^) + (0.1*P*)^2^ + 0.140*P*] where *P* = (*F*_o_^2^ + 2*F*_c_^2^)/3
1683 reflections	(Δ/σ)_max_ < 0.001
118 parameters	Δρ_max_ = 0.20 e Å^−^^3^
0 restraints	Δρ_min_ = −0.33 e Å^−^^3^

### Special details

**Table:**

Geometry. All esds (except the esd in the dihedral angle between two l.s. planes) are estimated using the full covariance matrix. The cell esds are taken into account individually in the estimation of esds in distances, angles and torsion angles; correlations between esds in cell parameters are only used when they are defined by crystal symmetry. An approximate (isotropic) treatment of cell esds is used for estimating esds involving l.s. planes.
Refinement. Refinement of F^2^ against ALL reflections. The weighted R-factor wR and goodness of fit S are based on F^2^, conventional R-factors R are based on F, with F set to zero for negative F^2^. The threshold expression of F^2^ > 2sigma(F^2^) is used only for calculating R-factors(gt) etc. and is not relevant to the choice of reflections for refinement. R-factors based on F^2^ are statistically about twice as large as those based on F, and R- factors based on ALL data will be even larger.

### Fractional atomic coordinates and isotropic or equivalent isotropic displacement parameters (Å^2^)

**Table:**

	*x*	*y*	*z*	*U*_iso_*/*U*_eq_	
S	0.75133 (11)	0.83172 (8)	0.57111 (9)	0.0414 (3)	
N	0.1391 (4)	0.1964 (3)	0.9738 (3)	0.0443 (6)	
O1	0.9506 (3)	0.7668 (3)	0.5545 (4)	0.0715 (8)	
C1	−0.0335 (5)	0.0843 (4)	1.0382 (4)	0.0479 (8)	
H1B	−0.1492	0.1292	1.0136	0.057*	
H1C	−0.0774	0.0735	1.1557	0.057*	
O2	0.7244 (4)	0.8981 (3)	0.4285 (3)	0.0532 (6)	
C2	0.1219 (4)	0.3116 (3)	0.8807 (3)	0.0410 (7)	
H2B	0.0013	0.3200	0.8601	0.049*	
C3	0.2822 (4)	0.4346 (3)	0.8017 (3)	0.0366 (6)	
C4	0.2457 (4)	0.5596 (4)	0.7043 (4)	0.0422 (7)	
H4A	0.1227	0.5624	0.6871	0.051*	
C5	0.3882 (4)	0.6802 (3)	0.6324 (3)	0.0405 (7)	
H5A	0.3619	0.7641	0.5677	0.049*	
C6	0.5702 (4)	0.6742 (3)	0.6579 (3)	0.0362 (6)	
C7	0.6120 (5)	0.5482 (4)	0.7525 (4)	0.0503 (8)	
H7A	0.7364	0.5441	0.7675	0.060*	
C8	0.4670 (5)	0.4292 (4)	0.8242 (4)	0.0472 (8)	
H8A	0.4937	0.3447	0.8881	0.057*	
C9	0.6747 (6)	0.9854 (4)	0.7201 (4)	0.0578 (9)	
H9A	0.7652	1.0772	0.6829	0.087*	
H9B	0.6811	0.9399	0.8195	0.087*	
H9C	0.5357	1.0231	0.7393	0.087*	

### Atomic displacement parameters (Å^2^)

**Table:**

	*U*^11^	*U*^22^	*U*^33^	*U*^12^	*U*^13^	*U*^23^
S	0.0377 (4)	0.0343 (4)	0.0478 (5)	−0.0113 (3)	−0.0116 (3)	0.0135 (3)
N	0.0499 (15)	0.0369 (13)	0.0412 (14)	−0.0182 (11)	−0.0110 (11)	0.0078 (11)
O1	0.0380 (13)	0.0554 (14)	0.111 (2)	−0.0117 (10)	−0.0194 (13)	0.0313 (14)
C1	0.0466 (17)	0.0404 (16)	0.0470 (17)	−0.0190 (13)	−0.0059 (13)	0.0091 (13)
O2	0.0648 (15)	0.0483 (12)	0.0440 (12)	−0.0185 (10)	−0.0178 (10)	0.0166 (10)
C2	0.0378 (15)	0.0369 (15)	0.0445 (16)	−0.0103 (12)	−0.0106 (13)	0.0008 (12)
C3	0.0399 (15)	0.0289 (13)	0.0362 (14)	−0.0081 (11)	−0.0086 (12)	0.0024 (11)
C4	0.0345 (15)	0.0385 (15)	0.0533 (18)	−0.0040 (12)	−0.0167 (13)	0.0079 (13)
C5	0.0419 (16)	0.0330 (14)	0.0455 (16)	−0.0032 (12)	−0.0160 (13)	0.0109 (12)
C6	0.0372 (15)	0.0300 (13)	0.0387 (14)	−0.0081 (11)	−0.0116 (12)	0.0078 (11)
C7	0.0437 (17)	0.0461 (17)	0.067 (2)	−0.0149 (13)	−0.0280 (15)	0.0225 (15)
C8	0.0528 (18)	0.0381 (15)	0.0572 (18)	−0.0143 (13)	−0.0287 (15)	0.0213 (13)
C9	0.068 (2)	0.0509 (19)	0.055 (2)	−0.0248 (16)	−0.0214 (17)	0.0071 (15)

### Geometric parameters (Å, °)

**Table:**

S—O1	1.426 (2)	C3—C4	1.384 (4)
S—O2	1.433 (2)	C4—C5	1.379 (4)
S—C9	1.755 (4)	C4—H4A	0.9300
S—C6	1.771 (3)	C5—C6	1.377 (4)
N—C2	1.254 (4)	C5—H5A	0.9300
N—C1	1.461 (3)	C6—C7	1.388 (4)
C1—C1^i^	1.513 (6)	C7—C8	1.380 (4)
C1—H1B	0.9700	C7—H7A	0.9300
C1—H1C	0.9700	C8—H8A	0.9300
C2—C3	1.476 (4)	C9—H9A	0.9600
C2—H2B	0.9300	C9—H9B	0.9600
C3—C8	1.382 (4)	C9—H9C	0.9600
			
O1—S—O2	118.21 (16)	C5—C4—H4A	119.4
O1—S—C9	108.72 (19)	C3—C4—H4A	119.4
O2—S—C9	108.48 (16)	C6—C5—C4	118.9 (3)
O1—S—C6	108.36 (14)	C6—C5—H5A	120.6
O2—S—C6	108.51 (14)	C4—C5—H5A	120.6
C9—S—C6	103.57 (15)	C5—C6—C7	121.0 (3)
C2—N—C1	116.2 (3)	C5—C6—S	119.4 (2)
N—C1—C1^i^	109.3 (3)	C7—C6—S	119.6 (2)
N—C1—H1B	109.8	C8—C7—C6	119.2 (3)
C1^i^—C1—H1B	109.8	C8—C7—H7A	120.4
N—C1—H1C	109.8	C6—C7—H7A	120.4
C1^i^—C1—H1C	109.8	C7—C8—C3	120.6 (3)
H1B—C1—H1C	108.3	C7—C8—H8A	119.7
N—C2—C3	123.8 (3)	C3—C8—H8A	119.7
N—C2—H2B	118.1	S—C9—H9A	109.5
C3—C2—H2B	118.1	S—C9—H9B	109.5
C8—C3—C4	119.1 (2)	H9A—C9—H9B	109.5
C8—C3—C2	121.7 (3)	S—C9—H9C	109.5
C4—C3—C2	119.2 (3)	H9A—C9—H9C	109.5
C5—C4—C3	121.2 (3)	H9B—C9—H9C	109.5
			
C2—N—C1—C1^i^	110.1 (4)	O2—S—C6—C5	−26.6 (3)
C1—N—C2—C3	−178.8 (3)	C9—S—C6—C5	88.5 (3)
N—C2—C3—C8	0.9 (5)	O1—S—C6—C7	24.9 (3)
N—C2—C3—C4	−178.3 (3)	O2—S—C6—C7	154.4 (3)
C8—C3—C4—C5	−1.3 (5)	C9—S—C6—C7	−90.4 (3)
C2—C3—C4—C5	177.9 (3)	C5—C6—C7—C8	−1.2 (5)
C3—C4—C5—C6	0.4 (4)	S—C6—C7—C8	177.8 (3)
C4—C5—C6—C7	0.8 (5)	C6—C7—C8—C3	0.3 (5)
C4—C5—C6—S	−178.2 (2)	C4—C3—C8—C7	0.9 (5)
O1—S—C6—C5	−156.1 (3)	C2—C3—C8—C7	−178.2 (3)

Symmetry codes: (i) −*x*, −*y*, −*z*+2.

### Hydrogen-bond geometry (Å, °)

**Table:**

*D*—H···*A*	*D*—H	H···*A*	*D*···*A*	*D*—H···*A*
C4—H4A···O1^ii^	0.93	2.52	3.241 (4)	135

Symmetry codes: (ii) *x*−1, *y*, *z*.
